# Effect of continuous positive airway pressure (CPAP) on mild-moderate obstructive sleep apnea (OSA) related dreaminess: A case report

**DOI:** 10.1097/MD.0000000000018949

**Published:** 2020-03-20

**Authors:** Pei Xue, Junying Zhou, Fei Lei, Lu Tan, Xiao Li, Xiangdong Tang

**Affiliations:** Sleep Medicine Center, Department of Respiratory and Critical Care Medicine, Mental Health Center, West China Hospital, Sichuan University, Chengdu, China.

**Keywords:** continuous positive airway pressure, dreaminess, obstructive sleep apnea, polysomnography

## Abstract

**Introduction:**

Dreaminess is one of the common symptoms of sleep disorders and often leads to complaint of poor sleep quality and morning fatigue. Literatures on the relationship between obstructive sleep apnea (OSA) and dreams have been reported with contradictory results. In this case report, we identified a moderate OSA related dreaminess that was successfully treated by continuous positive airway pressure (CPAP).

**Patient concerns:**

We present a case of a 47-year-old woman who was bothered by all-night dreaminess for over 20 years.

**Diagnosis:**

An overnight polysomnography (PSG) examination showed the apnea-hypopnea index (AHI) was 21.7 events/hour and the rapid eye movement (REM)-AHI was 46.3 events/hour. The patient was diagnosed with moderate OSA.

**Interventions:**

The patient received auto CPAP therapy.

**Outcomes:**

The symptoms of dreaminess and daytime functioning significantly improved after CPAP treatment. During the 4-month follow up, 3 CPAP titrations showed that OSA events and OSA related REM interruption almost disappeared. On the night of PSG diagnosis, only 1 non-rapid eye movement sleep 3 (N3) episode occurred before the first REM episode. Nevertheless, N3 episodes were observed before the majority of REM episodes on all three nights of CPAP titration.

**Conclusion:**

This case suggests that specific REM related OSA could be the main reason for dreaminess symptoms and could be successfully treated by CPAP. The identification of OSA, especially for mild-moderate OSA, has not received enough attention in the management of complaints of dissatisfactory sleep issues. We believe this case has educational value in clinical practice.

## 1 Introduction

Dreaminess is one of the common symptoms of sleep disorders, which is often associated with poor sleep quality and even morning fatigue. Obstructive sleep apnea (OSA) is one of the most common sleep disorders, which is characterized by repetitive obstruction of the upper airway, intermittent hypoxemia, sleep fragmentation, and increased daytime fatigue and sleepiness, and the risk of cardiovascular disease.^[1]^ Literatures on the relationship between OSA and dreams have been reported with contradictory results. Less dream recall in patients with OSA has been reported by some investigators. However, more dreams with emotional content, particularly violent and aggressive dreams, in OSA patients were found by others.^[2–5]^ Previous study also found that severe sleep apnea can present with dream enacting behaviors and unpleasant dreams and could be eliminated with the therapy of continuous positive airway pressure (CPAP).^[6]^ In a follow-up study on patients with severe OSA, Carrasco et al also demonstrated a decrease in the dream recall rate during the first night of CPAP therapy as well as the next 2-year CPAP treatment.^[3]^

However, there is little evidence on the effect of CPAP on mild-moderate OSA related dreaminess. Here we reported a case in which CPAP was successfully used to treat the patient who was bothered by dreaminess for the past 2 decades.

## 2 Case report

A 47-year-old woman presented to our sleep medicine clinic with a chief complaint of dreaminess during the whole night for the past 2 decades. She was troubled by dreams characterized by strong negative emotions which sometimes resulted in non-refreshing sleep and bad mood. She felt tired in the daytime and was anxious about the poor sleep quality. In recent 6 to 7 years, her dreaminess symptoms gradually worsened and she began to seek for medical assistance from physicians, psychiatrists, and neurologists for many times. However, no confirmed diagnosis was made. Some physicians suggested her taking low-dose sleeping pills if the symptom really affected her life, but she did not take any drugs in fear of possible side effects. On learning about our sleep medicine center from her friends, she came for medical assistant on July 17th, 2017. Her body mass index was 21.23 kg/m^2^. No history of mental disorders or neuropsychiatric disorders such as depression, anxiety, or post-traumatic stress disorder (PTSD) was identified. Neurological and mental examinations were unremarkable. No special findings were obtained in the routine medical examination or laboratory results. Because she reported snoring during sleep, we scheduled an overnight polysomnography (PSG) examination for her.

**Figure 1:**
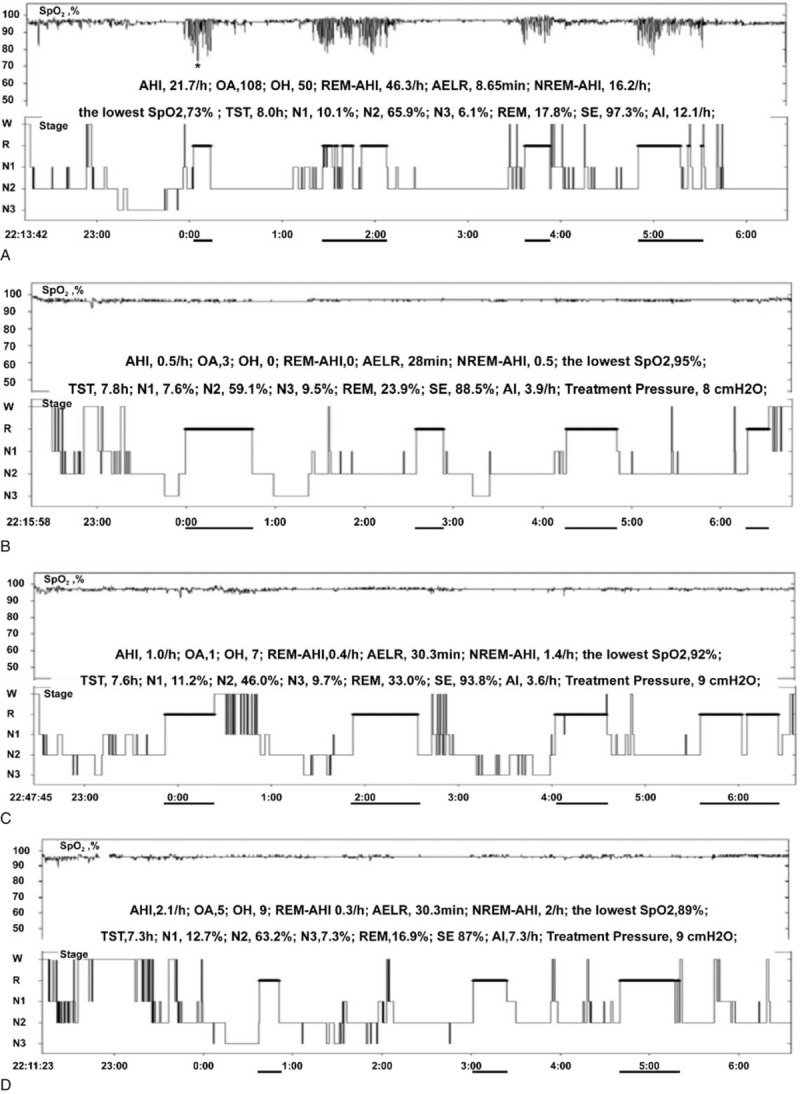
Changes of histogram of sleep determined by PSG. A Night of diagnosis. B First CPAP titration night (4 days after diagnosis). C Second CPAP titration night (65 days after diagnosis). D Third CPAP titration night (123 days after diagnosis). Lines under the *x*-axis indicate episodes of rapid eye movement sleep cluster. ∗ indicates the lowest SpO_2_. AELR = average episode length of REM sleep, AHI = apnea/hypopnea index, AI = arousal index, CPAP = continuous positive airway pressure, N1–3 = non-rapid eye movement sleep 1–3, NREM = non-rapid eye movement sleep, OA = number of obstructive apneas, OH = number of obstructive hypopneas, PSG = polysomnography, REM = rapid eye movement sleep, SE = sleep efficiency, SpO_2_ = capillary oxygen saturation, TST = total sleep time.

The PSG examination showed that she had a total sleep time (TST) of 8.0 hour with apnea-hypopnea index (AHI) of 21.7 events/hour. There were 108 events of obstructive apnea (the longest apnea 86 seconds) and 106 events of hypopnea. The sleep architecture was discontinuous because of the episodes of apneas and hypopnea. Rapid eye movement (REM) sleep was 17.8% and non-rapid eye movement sleep 3 (N3) was 6.1% during the night. As shown in Fig. 1A, apneas and hypopneas were particularly prominent during REM sleep (REM-AHI was 46.3/hour). Apnea events and apnea related falls in oxygen saturation were the majority in REM sleep but not during non-rapid eye movement (NREM) sleep (NREM-AHI was 16.2/hour); the lowest peripheral capillary oxygen saturation (73%) and the longest apnea (86 seconds) were also during REM sleep. We successively named REM periods in the Fig. 1A as REM cluster I to IV. The REM episodes were interrupted by OSA events, particularly in II REM cluster (Fig. 1A). When examining her PSG results more closely, we observed 17 respiratory arousals during total REM sleep time (86.5 minutes) and 15 during total NREM sleep time (393.1 minutes). The patient complained that she had similar dreaminess symptoms and dissatisfactory sleep to before.

Four days later, the patient was scheduled to receive CPAP titration. As shown in Fig. 1B, she had 7.8 hours of TST and AHI was successfully reduced to 0.5/hours. The CPAP pressure was 8 cm H_2_O. REM was 23.9% and N3 was 9.5%. Overnight sleep architecture, particular REM sleep, became much more consolidated. The arousal numbers decreased (from 97 to 31) whereas the average episode length of REM sleep increased (from 8.65 to 28). Moreover, the patient reported that she did not seem to dream all the night and had much better perceived overnight sleep as well as daytime function.

Then she began to receive auto CPAP therapy (Somnobalance e). In order to know about the objective sleep valuations, we arranged another two CPAP titrations in the second month (Fig. 1C) and the fourth month (Fig. 1D) after CPAP therapy, respectively. Two-night PSG results were similar to that of the initial CPAP titration night, apnea and hypopnea almost disappeared, and the sleep architecture and REM-sleep continuity both became much more stable. The pressures both were 9 cm H_2_O in the 2 CPAP titrations. The numbers of awakenings and arousals during REM sleep both reduced. Compared with the first 2 CPAP titration nights, the patient complained that she had unusually uncomfortable sleep because of backache on the third CPAP titration night.

**Figure 2:**
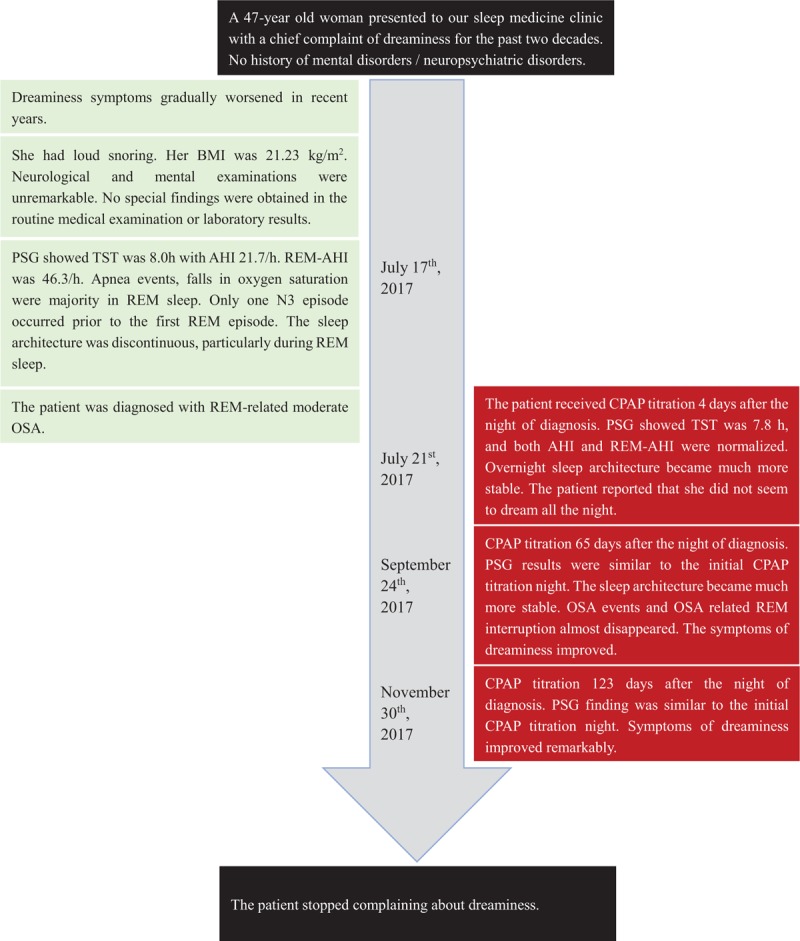
The timeline of clinical history. AHI = apnea-hypopnea index, BMI = body mass index, CPAP = continuous positive airway pressure, OSA = obstructive sleep apnea, PSG = polysomnography, REM = rapid eye movement sleep, TST = total sleep time.

During the follow-up days, the patient reported that the symptoms of dreaminess were gradually alleviated, and the frequency of dream recall reduced from more than 3 times per night to a few times per month. Moreover, the dream contents were more positive after CPAP treatment. She was satisfied with the sleep quality and felt more energetic and motivated during the daytime. Further examinations just one N3 sleep preceding REM sleep during the diagnostic PSG night but not during the rest of the nights. In all three titration nights, however, almost every REM episode was preceded by the N3 stage although the result of the third titration night was not very satisfactory because of the backache (Fig. 2). The comparison of qualitative and quantitative parameters before and after the CPAP therapy was presented in Table 1.

**Table 1:**
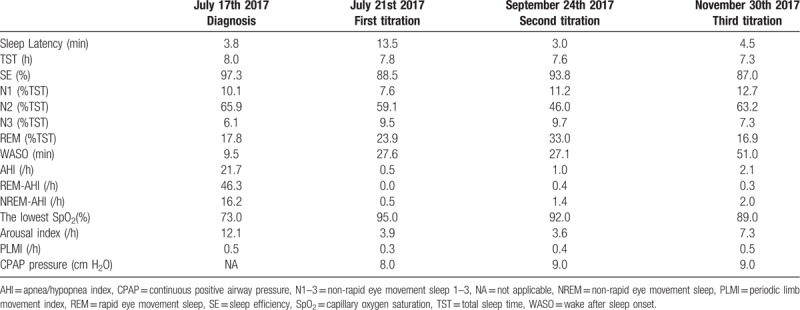
The comparison of sleep parameters before and after the CPAP therapy.

Due to the improvement of dreaminess symptoms and sleep quality, the patient had good compliance of using CPAP. Both the subjective sleep quality and objective sleep parameters were significantly improved following CPAP therapy. CPAP memory indicated the use of CPAP on 86% of the nights for an average of 5.4 hours per night, and the highest pressure was 8 cm H_2_O. Written informed consent was obtained from the participant for publication of this case report. A copy of the written consent is available for editorial review.

## 3 Discussion

Here we present a typical case in which CPAP was successfully used to treat dreaminess in a patient diagnosed with moderate OSA. Dreaming is a normal activity, which occurs mostly during REM sleep and results in dream recollection.^[7]^ However, all-night dreaminess is a major problem which results in poor sleep quality and severely affects daytime function, which was similar in this patient who was continually bothered by dreaminess throughout the past 2 decades. Researchers have pointed out that dreaminess can be the major impact factor of a person's morning mood.^[8]^ Besides, dreams not only affect emotions, but also influence decision making and routine work.^[9,10]^ Recently, a study reported that dreams might have relation to suicidality in normal adolescent population.^[11]^

Dreaminess as a nonspecific symptom of poor sleep quality is a common complaint to seek help in sleep clinic. However, the cause of dreaminess is still unclear. This patient was diagnosed with moderate OSA and her REM-AHI was 46.3/hour. The sleep architecture was interrupted by frequent respiratory events and respiratory-induced arousals, especially during REM sleep. For her, dreaminess symptoms existed constantly over the past 2 decades and were significantly improved after CPAP treatment, which indicated that it was indeed a case of OSA related dreaminess. In fact, complaints of dreaminess are frequent among OSA patients. To our knowledge, there are several factors that may influence dreaming in OSA. First, sleep fragmentation and repeated arousals of OSA are associated with high percentages of light sleep which may contribute to the awareness of the dreaming process and increase dream recall.^[12]^ Second, hypoxia and sleep fragmentation may be connected with cognitive dysfunction,^[13]^ which may worsen dream recall. In a longitudinal study, BaHammam et al found that nightmares in OSA patients were associated with a higher REM-AHI,^[14]^ and CPAP therapy significantly reduced nightmare occurrences. Although some researches have been done on the application of CPAP to improve OAS-related nightmares, its effects on dreaminess remain largely unknown. Gross et al^[15]^ found that patients with OSA had a tendency to dream less when they received effective CPAP therapy. This finding may be attributed to 2 things. For one thing, dreaminess symptoms were alleviated as a result of improvement on sleep fragmentation; for another, the increase in the amount of N3 sleep induced by CPAP increased the chance for N3 sleep to precede REM sleep. Because N3 sleep had low arousability, dream recall may become less efficient in REM sleep episode which occurs after the N3 sleep.^[12]^ In this regard, CPAP improves dreaminess symptoms via not only improving the continuity of the sleep architecture, but also increasing the chance for N3 sleep to precede REM.

In clinical practice, neurologists and psychiatrists generally use pharmacologic therapy featuring hypnotic medication as a common treatment option for dreaminess rather than explore the possible reason accounting for the symptoms. Whether the patients have OSA related symptoms (eg, loud snoring and witness apnea) are not routinely questioned. However, because the use of benzodiazepine and non-benzodiazepine sleeping aids may aggravate OSA,^[16]^ prescribing sleeping aids in dreaminess patients with risk factors for OSA may be associated with adverse effects.

## 4 Conclusions

Two lessons could be learned from this case. First, we should pay close attention to dreaminess symptoms once they become the main reason for medical treatment. Moreover, factors like snoring,^[17]^ sleep-disordered breathing, especially REM-related OSA should be considered as possible reasons for dreaminess. Second, reduced symptoms of dreaminess may be associated with better compliance to CPAP therapy. Evidence shows that higher sleep quality on the night of CPAP titration predicts future compliance with CPAP.^[18]^ Although CPAP therapy is the most effective and commonly prescribed treatment for OSA, poor compliance to CPAP resulted in negative health consequences. This suggests that improving CPAP adherence is a major public health concern. As previous studies have already demonstrated, about 46% to 83% patients reported using CPAP for fewer than 4 hours per night.^[19]^ We believe that the adherence to CPAP therapy could be increased by the therapy of dreaminess and OSA. That is, understanding the potential effects of CPAP therapy on dreaminess will be important for the successful long-term disease management of OSA.

## Author contributions

**Conceptualization:** Xiangdong Tang.

**Data curation:** Pei Xue, Xiao Li.

**Methodology:** Pei Xue, Fei Lei, Lu Tan.

**Writing – original draft:** Pei Xue.

**Writing – review & editing:** Junying Zhou, Xiangdong Tang.
